# Blood metabolite markers of neocortical amyloid-β burden: discovery and enrichment using candidate proteins

**DOI:** 10.1038/tp.2015.205

**Published:** 2016-01-26

**Authors:** N Voyle, M Kim, P Proitsi, N J Ashton, A L Baird, C Bazenet, A Hye, S Westwood, R Chung, M Ward, G D Rabinovici, S Lovestone, G Breen, C Legido-Quigley, R J B Dobson, S J Kiddle

**Affiliations:** 1 MRC Social, Genetic and Developmental Psychiatry Centre, Institute of Psychiatry, Psychology and Neuroscience, King's College London, London, UK; 2 Institute of Pharmaceutical Science, Kings College London, London, UK; 3 Department of Old Age Psychiatry, Institute of Psychiatry, Psychology and Neuroscience, King's College London, London, UK; 4 NIHR Biomedical Research Centre for Mental Health and Biomedical Research Unit for Dementia at South London and Maudsley NHS Foundation, London, UK; 5 Department of Psychiatry, University of Oxford, Warneford Hospital, Oxford, UK; 6 Proteomics Facility, Institute of Psychiatry, Psychology and Neuroscience, King's College London, London, UK; 7 Memory and Aging Center, University of California, San Francisco, San Francisco, CA, USA

## Abstract

We believe this is the first study to investigate associations between blood metabolites and neocortical amyloid burden (NAB) in the search for a blood-based biomarker for Alzheimer's disease (AD). Further, we present the first multi-modal analysis of blood markers in this field. We used blood plasma samples from 91 subjects enrolled in the University of California, San Francisco Alzheimer's Disease Research Centre. Non-targeted metabolomic analysis was used to look for associations with NAB using both single and multiple metabolic feature models. Five metabolic features identified subjects with high NAB, with 72% accuracy. We were able to putatively identify four metabolites from this panel and improve the model further by adding fibrinogen gamma chain protein measures (accuracy=79%). One of the five metabolic features was studied in the Alzheimer's Disease Neuroimaging Initiative cohort, but results were inconclusive. If replicated in larger, independent studies, these metabolic features and proteins could form the basis of a blood test with potential for enrichment of amyloid pathology in anti-amyloid trials.

## Introduction

The most common form of dementia is Alzheimer's disease (AD), a neurodegenerative condition that leads to severe cognitive impairment in later life. Currently, the disease mechanism of AD is not comprehensively understood, and consequently no disease-modifying treatments are available. Unfortunately, current symptomatic treatments only have a moderate effect.^1^ There is therefore a desperate need for a disease-modifying treatment for AD.

A definitive AD diagnosis can only be made post-mortem; however, neuropathological biomarkers (amyloid-β (Aβ) plaques and phosphorylated tau tangles) can be used to help differentiate AD from other dementias during a person's lifetime. These can be used in a clinical trial setting to ensure that all recruited participants have evidence of the target pathology. In a trial of Bapineuzumab, an anti-amyloid therapeutic, 14% of subjects had low amyloid. It was therefore unlikely that these subjects would see any benefit from the treatment. Furthermore, their involvement in that study would have reduced the statistical power of finding a treatment effect.^2^ Many trials now test for elevated neocortical amyloid burden (NAB) as an eligibility requirement.

Elevated NAB is also becoming an eligibility criterion for some prevention trials, such as the A4 trial.^3^ This trial aims to assess whether anti-amyloid therapeutics can delay early cognitive decline in asymptomatic individuals, a concept that has developed as a result of research showing that AD has a long prodromal stage.^4, 5^ The characteristic disease pathology of AD can begin to develop up to 20 years before any clinical symptoms.^6, 7^ This provides a window of time for a potential treatment to stop, or at least slow down, future progression of the disease.

Neuropathological biomarkers are measured by quantifying the concentrations of Aβ, tau and phosphorylated tau in the cerebrospinal fluid (CSF) or via positron emission tomography (PET) imaging. In addition, metabolites in CSF have been studied as possible biomarkers for AD and related phenotypes.^8^ However, the methods used to capture this information are invasive, require specialized equipment and are often expensive and hence impractical on a large scale.

Consequently, there is a high demand for a blood-based biomarker of AD that would be easier and potentially cheaper to attain.^9^ Metabolites are typically smaller than other biological molecules, and therefore have a greater chance of passing through a possibly weakened blood–brain barrier.^10^ This increases the chance that blood metabolites could serve as a biomarker of AD. A review of AD biofluid metabolite studies has highlighted sphingolipid and glutamate metabolism as being altered in AD, besides the metabolism of molecules with antioxidant properties.^11^ In addition, Proitsi *et al.* used a case–control study design to discover a set of long-chain cholesteryl esters associated with AD, whereas other studies have aimed to predict conversion from mild cognitive impairment (MCI) to AD.^12, 13, 14^ Mapstone *et al.*^13^ discovered a lipid panel from peripheral blood that predicted conversion from control status to amnestic MCI or AD with 90% accuracy. The panel highlighted metabolites involved in cell membrane integrity and lipids involved in cell signaling, as also suggested by Whiley *et al.*^15^

These studies should now be extended to identify markers of amyloid pathology. Such markers could then be used to enrich clinical trials with elevated NAB as an eligibility criterion. Using a blood test as a filter before a confirmatory lumbar puncture or PET scan could improve the efficiency of clinical trials by reducing the cost of recruitment.^16^

Analogous approaches have already been applied to identify genetic and protein biomarkers of NAB. A polygenic risk score trained on AD diagnosis has been shown to associate with CSF Aβ levels in the Alzheimer's Disease Neuroimaging Initiative (ADNI) cohort.^17^ Similarly, multiple studies have identified potential blood protein biomarkers of NAB, as reviewed in Voyle *et al.*^18^ Of particular interest are replicated markers of NAB including pancreatic polypeptide (PPY) and fibrinogen gamma chain (FGG).^16, 18^

This is the first study to investigate associations between blood metabolites and NAB. Further, we present the first multi-modal analysis of blood markers in AD biomarker discovery. We consider whether a blood metabolite signal complements that of previously discovered blood protein biomarkers of NAB.

## Materials and methods

### Cohorts

#### UCSF

Subjects were recruited from those enrolled in the University of California, San Francisco (UCSF) AD Research Centre. Study information has been given elsewhere.^19, 20^ The study was approved by the UCSF and Lawrence Berkeley National Laboratory committees for human research. All subjects provided written informed consent before participating.

#### ADNI

ADNI is a longitudinal cohort study aiming to validate the use of biomarkers in AD clinical trials and diagnosis. Data used in the preparation of this article were obtained from the ADNI database (adni.loni.usc.edu). The ADNI was launched in 2003 as a public–private partnership, led by Principal Investigator Michael W Weiner. The primary goal of ADNI has been to test whether biological markers and clinical and neuropsychological assessment can be combined to measure the progression of MCI and AD. For information, see www.adni-info.org. ADNI was approved by the institutional review boards of all participating institutions, and written informed consent was obtained from all participants.

### Metabolomics

#### UCSF

Blood plasma samples were available for 91 subjects enrolled in the UCSF AD Research Centre. The ultra performance liquid chromatography-tandem mass spectrometry (UPLC-MS/MS) method used in this study has been previously published.^15^ Twenty microliters of plasma per subject was required for analysis, with sample treatment being described elsewhere.^15, 21^ The method primarily detects lipids and has been shown to measure abundances of over 4500 metabolic features. The instruments included a Waters ACQUITY UPLC and Xevo Quadrupole Time-of-flight System (Waters, Milford, CT, USA). The Xevo Quadrupole Time-of-flight System was operated in both negative and positive ion modes. Samples were analyzed as one batch in a randomized order, with pooled plasma quality-control (QC) samples run between every 10 samples.

#### ADNI

Metabolite data were available for 853 blood serum samples. Twenty-four subjects had two samples included in the study. Targeted metabolomics analysis was performed using the AbsoluteIDQ p180 assay (Biocrates Life Sciences, Innsbruck, Austria) requiring 10 μl of serum per sample. The samples were run in 11 batches with two pooled QC samples present in each batch: one run before the samples and one afterward. More information on the assay, sample treatment and instruments can be downloaded from the ADNI website (adni.loni.usc.edu/).

### Candidate protein assays (UCSF only)

The proteomics approach used in this study has been described elsewhere.^16^ In short, a set of candidate proteins was quantified using single analyte sandwich enzyme-linked immunosorbent assays. In this study we investigated two proteins that have been replicated as NAB markers: FGG and PPY.^16, 18, 22, 23^

### NAB measurements

#### UCSF

Details of PET imaging are given elsewhere.^20^ All PET scans used Pittsburgh compound B (11C-PiB) as the radioactive tracer. Scans were performed using two different scanner types, Biograph TruePoint 6 PET/computed tomography (*N*=9) and Siemens ECAT EXACT HR PET (*N*=69), and were processed using methods described by Lehmann *et al.*^24^

We considered two PET outcomes. Two experienced raters who were blinded to plasma and clinical data rated the scans as either high NAB or low NAB to give a dichotomous outcome. Second, the 50–70-min standardized uptake value ratio (SUVR) was used as a continuous outcome.^25^

#### ADNI

Details of PET imaging in ADNI (using both PiB and AV45 markers) and CSF measurements are detailed elsewhere (www.adni-info.org). PET end points were dichotomized into high and low NAB at the SUVR thresholds previously used in ADNI (1.5 for PiB and 1.11 for AV45). CSF measures of amyloid were taken from the data set ‘UPENNBIOMK2' available on the ADNI website. The CSF measures were dichotomized at the previously published threshold (192 pg ml^−1^). We combined the three amyloid end points into a combined amyloid end point to maximize sample size. A subject was classified as NAB-positive if at least one measurement indicated high brain amyloid burden, and classified as NAB-negative otherwise.

### Statistical analysis

All statistical analyses were performed in R version 3.1.1.^26^

### Data pre-processing

In UCSF, metabolic feature data were extracted from netCDF files using the R package ‘XCMS'.^27^ The package performed filtration and peak identification before matching peaks across samples and performing a retention time correction. Following data extraction, the negative- and positive-mode data were processed separately using the pipeline detailed in Supplementary Text 1. ADNI data were also processed using this pipeline. The processing included outlier removal, normalization through autoscaling and a log base 2 transformation as well as batch correction using the empirical Bayes method, ComBat.^28^

After pre-processing, the UCSF data collected in negative and positive modes were merged.

Protein data were subject to a natural logarithm transformation and screened for per sample, per protein outliers defined as values outside of 6 s.d.'s of the mean (as above). Each protein was autoscaled.

### Single metabolic feature analysis

Single metabolic feature analysis was performed in UCSF for both NAB outcomes for each of the 2760 metabolic features detected. SUVR was linearly regressed against each metabolic feature in turn with *APOE* ɛ4 status and age included as covariates in the model. The *APOE* ɛ4 status is defined as 1 if a subject's genotype contained any ɛ4 alleles and 0 otherwise. Similarly, logistic regression was performed for the dichotomous outcome. In both cases, a Benjamini–Hochberg correction of the false discovery rate was applied.

### Multiple metabolic feature analysis

Multiple metabolic feature analysis was performed on UCSF data using the R package ‘caret'.^29^ Partial least squares (PLS) and PLS discriminant analysis were used for the continuous and dichotomized outcomes, respectively. Ideally, we would have split the data into a training and test set; however, owing to relatively small sample size, this was not possible and a cross-validation (CV) approach was taken instead. All metabolic features, age and *APOE* ɛ4 status were included in the model building. The number of components to include was tuned using five-fold CV through the ‘train' function. Recursive feature elimination was used to select a subset of variables using five-fold CV. The subset sizes considered varied from 2 to 99 in steps of 1 and from 100 to the total number of covariates in steps of 100. In PLS modeling, the lowest root mean squared error (RMSE) was used to select the best model, whereas for PLS discriminant analysis the highest accuracy was used. The function ‘pickSizeTolerance' was then applied in an attempt to find a smaller subset of variables that maintained RMSE or accuracy to within 5% of the best model. We also built models using the 10 most important predictors, the maximum number of metabolic features we could feasibly identify. Model statistics resulting from five times CV within recursive feature elimination are presented in this report.

For comparison, we used five-fold CV to build a model based on age and *APOE* ɛ4 status alone using the ‘train' function to tune the number of components as above. This method was used, despite the small number of predictors, to ensure continuity between modeling techniques. We checked that the results were consistent with those gained from a linear regression model. This model is referred to as the demographic-only model throughout.

### Metabolic feature and protein joint analysis

The final multiple metabolic feature models were updated by adding proteins. Model building followed that of the demographic-only model detailed above. FGG and PPY were included both together and separately. We also modeled PPY and FGG (with and without age and *APOE* ɛ4 status) against continuous and dichotomized NAB without metabolic features for comparison.

### Putative metabolite identification

Putative identification of selected metabolic features from statistical analysis was attempted using the median *m/z* and their corresponding retention time, initially using an in-house database and the Human Metabolome Database.^21, 30^ To enable the confirmation of features from the database-matching, fragmentation patterns were analyzed using level-two MS spectra.

### Replication in ADNI

We searched the ADNI metabolite data for any of the metabolic features putatively identified in UCSF. Logistic regression models of the combined amyloid end point were built using individual metabolites as predictors, covarying for age and *APOE* ɛ4 status.

### Code availability

All R codes used to generate this analysis are available from the corresponding author on request.

## Results

### Data pre-processing

#### UCSF

The R package ‘XCMS' extracted data for 248 metabolic features from negative ionization mode and 2807 metabolic features from positive ionization mode. We ran UPLC-MS/MS in the positive mode on 91 subject samples and 11 pooled QC samples. In the negative mode, data were available for 90 samples and 10 pooled QC samples.

#### ADNI

Data were available for 141 metabolites in 853 samples. This included 22 pooled QC samples and 24 replicates. As no documentation of technical replicates was given by ADNI, the first value was taken. This reduced the sample size to 829, including the 22 QC samples.

**Figure 1:**
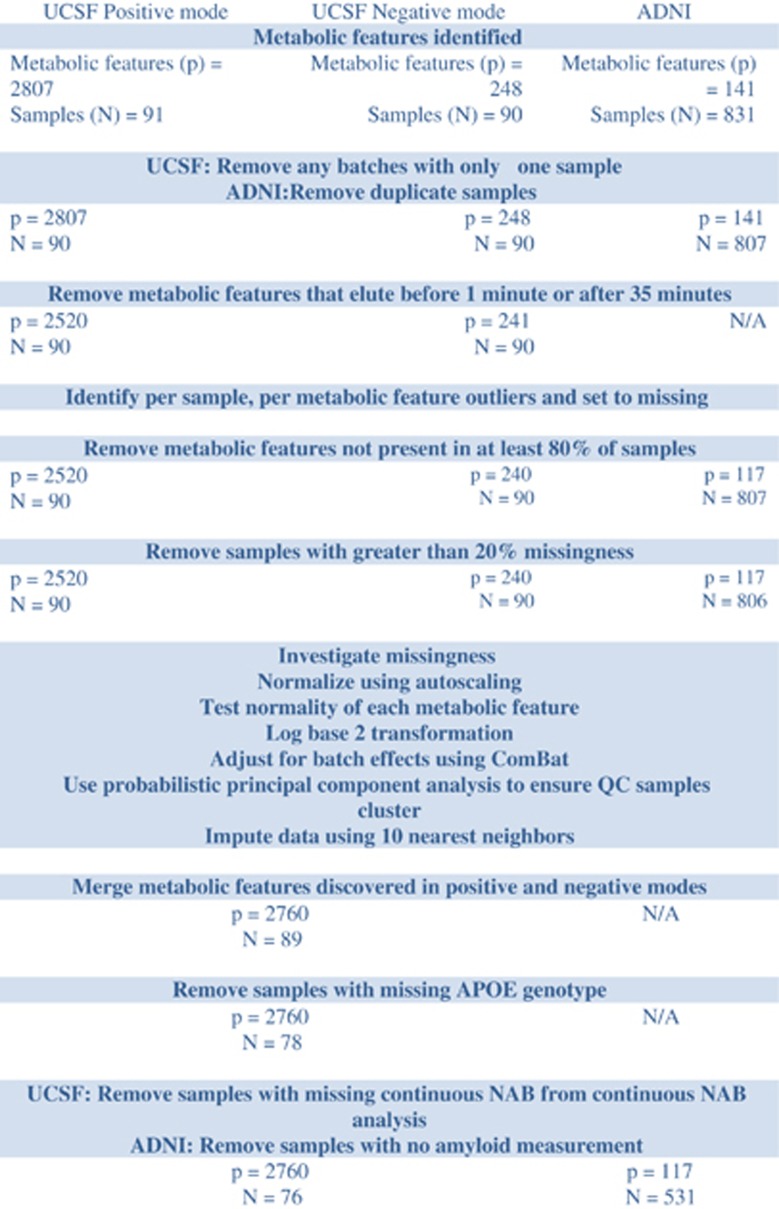
Overview of pre-processing steps affecting the number of metabolic features and samples.

Figure 1 gives an overview of the pre-processing steps. In UCSF, this processing resulted in 78 subjects with dichotomous NAB and 76 subjects with continuous NAB. We had a total of 2760 metabolic features: 240 from the negative mode and 2520 from the positive mode. In ADNI, the processing resulted in 531 subjects with the combined amyloid end point and 116 metabolic features.

### Cohort demographics

An overview of demographics for subjects included in the dichotomous NAB analysis is given in Table 1. The subjects used here have a wide range of diagnoses that can be grouped into four categories: AD, fronto-temporal dementia, MCI and healthy controls. Of these 78 subjects, 2 did not have SUVR available, reducing the number of subjects in the continuous analysis to 76. The demographics of this subpopulation are given in Supplementary Table 1. It is important to note that the population is relatively balanced in terms of age and scanner type between high and low NAB groups.

An overview of demographics for subjects included in the ADNI replication analysis is also shown in Table 1.

### Single metabolic feature analysis

For both continuous and dichotomized NAB, no metabolic features passed a *q*-value threshold of 0.1. Supplementary Tables 2 and 3 give full results.

### Multiple metabolic feature analysis

#### Continuous NAB

The multiple metabolite model with the lowest error was found for 100 predictors, all of which were metabolic features (CV RMSE=0.53, CV *R*^2^=0.10). A tolerance set was generated to maintain error (that is, CV RMSE) within 5% of the value achieved by the optimal model (0.53). The reduced model contained 17 of these metabolic features (CV RMSE=0.55, CV *R*^2^=0.07).

The 10 predictor models contained only one component (CV RMSE=0.56, CV *R*^2^=0.05). Cross-validated model statistics are given in Table 2, illustrating that the models including metabolic features do not outperform age and *APOE* in this training data. For information on the metabolic features included in the final models see Table 3.

Addition of the proteins FGG and PPY to the 17-metabolic-feature model increased cross-validated *R*^2^ to 0.57, explaining more variation in NAB than metabolic features or proteins alone (*R*^2^=0.07 and 0.21, respectively).

#### Dichotomized NAB

The best model and tolerance set model were the same, both containing five-metabolic-feature predictors (CV accuracy=0.72, CV sensitivity=0.65, CV specificity=0.76). Model statistics for the final models are given in Table 2. We see an improved accuracy of 72% compared with age and *APOE* alone at 58%. For information on the five metabolic features included in the final model see Table 3.

The addition of the protein FGG to the five-metabolic-feature model increased accuracy to 79%, with sensitivity and specificity both above 70% (71% and 84%, respectively). The two protein models (FGG and PPY only) gave an identical accuracy to the five-metabolic-feature model at 72%, driven by a high specificity (93%).

#### Putative metabolite identification

**Figure 2:**
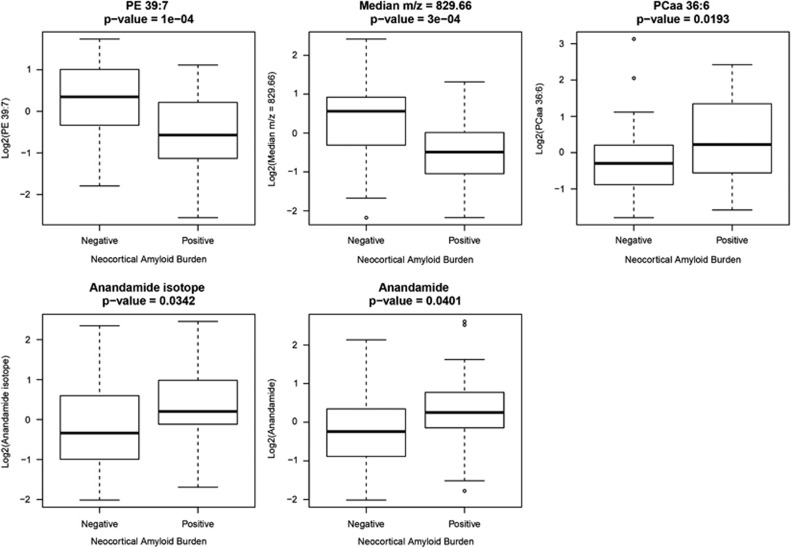
Boxplots showing metabolic feature levels between high and low neocortical amyloid burden (NAB) groups for the five metabolic features included in the final model of dichotomized NAB. Student's *t-*test was used to generate a *P*-value.

We aimed to putatively identify the five metabolic features that were included in the final model of dichotomized NAB (Figure 2). We were able to identify four of these five metabolic features. No suitable surrogate metabolic feature was available for the unidentified metabolite.

One of the four metabolic features was discovered in negative-mode UPLC-MS/MS (median *m/z*=775.68) and has been identified as a phosphatidylethanolamine (PE 39:7). The remaining metabolic features were discovered in the positive mode. The metabolic feature with median *m/z*=647.59 and an isotope (median *m/z*=648.59) are likely to be anandamide (linoleoyl ethanolamide (2M+H)).^31^ As expected, these isotopes are highly correlated (Pearson's correlation coefficient=0.966). Fragmentation patterns of the metabolic feature with the median *m/z*=778.63 suggest a phosphatidylcholine (PCaa 36:6).

#### Replication in ADNI

One of the four putatively identified metabolites from UCSF was found in the ADNI data: PCaa 36:6. In the logistic regression model of the combined amyloid end point, PCaa 36:6 had an estimate of −0.729 (*P*=0.066).

## Discussion

To the best of our knowledge, this is the first study to investigate associations between blood metabolites and amyloid burden in the brain. We have used non-targeted metabolomics to predict NAB in subjects from the UCSF AD research center. We also present the first analysis to combine protein and metabolite data in the search for a biomarker for AD.

We found a panel of five metabolic features that predicted amyloid positivity with an accuracy of 72%. If the model specificity (76%) seen here is maintained in a replication study, it could be useful in a screening setting where a large proportion of subjects would have high amyloid burden. As the metabolite panel correctly identified subjects with low amyloid levels, 76% of the time it could be useful in reducing the number of patients with low amyloid burden unnecessarily subjected to further procedures. Interestingly, no metabolic feature model retained age or *APOE* ɛ4 status. This population appeared relatively balanced with respect to these two variables, possibly accounting for the lack of inclusion. Alternatively, effects of age and *APOE*, which are well known to be associated with amyloid burden, could be accounted for in surrogate metabolic feature variables.^6, 7^ Analysis of single metabolic features gave no significant results. However, low statistical power in the current study means that this approach should not be ruled out in further study of larger cohorts.

We were able to putatively identify four of the five metabolic features included in the model of dichotomized NAB. Of those identified, one was a phosphatidylcholine compound (PCaa 36:6). PCs are a group of compounds previously implicated in AD by Whiley *et al.* and others.^12, 15^ In particular, PCaa 36:6 was included in the 10-lipid panel suggested by Mapstone *et al.* to predict conversion to amnestic MCI or AD with 90% accuracy. The association is in the opposite direction to that seen here, which could be explained by differences in disease stage between the cohorts. PCs are phospholipids that form a substantial component of biological membranes, and in this study show increased abundance in subjects with high NAB. Chung *et al.* state that PCs improve memory in mouse models, corresponding with the direction of association seen by Mapstone *et al.*^13^ However, a Cochrane review has surmised that there is not sufficient evidence to extend this conclusion to humans.^33^

We were able to test associations of PCaa 36:6 in the ADNI cohort. We saw a direction of association concurrent with that seen by Mapstone *et al.* but opposite to that seen in the UCSF cohort.^32^ Subjects in the ADNI cohort are diagnostically more similar to those used by Mapstone *et al.*, which could account for this similarity. Further, as we could only test the one metabolite, it is possible that this discrepancy is because we could not include the other four metabolites. ADNI is currently the only other cohort that has both metabolite and amyloid data available, and consequently this is the maximum extent of replication we can perform. It is essential that further attempts at replication are made in larger, independent studies.

We were also able to identify a PE (39:7). PEs are also a subtype of phospholipids that can be found in biological membranes. Interestingly, in humans they are largely found in tissues of the central nervous system and when methylated yield phosphatidylcholines.^34^ PEs are also implicated in prion disease, where they cause aggregation of the prion protein.^35^ In this study PE 39:7 was reduced in subjects with high NAB. PEs are also substrates for the synthesis of the final metabolic feature we were able to identify: anandamide.^36^ Anandamide is an endogenous cannabinoid neurotransmitter that, on connection with receptors in the cell membrane, reduces the release of other neurotransmitters in the brain.^37^ Anandamide is fat soluble, allowing it to pass through the blood–brain barrier and is made in areas of the brain important in memory. It is hypothesized that anandamide is involved in the creation and deletion of short-term connections between nerve cells.^38^ In support of this theory, the presence of anandamide has been shown to impair memory in rats.^38^ In this study anandamide is reduced in subjects with high NAB. This supports findings by Jung *et al.*^39^ who see an Aβ-dependent association of anandamide with cognitive decline in samples of brain tissue.

This study shows for the first time that the addition of candidate proteins (FGG and PPY) to metabolic feature models improves results. These results are promising and warrant further study while reinforcing the idea that a multi-modal approach may be more effective in AD biomarker discovery than single modality approaches.

Although the results we present here are interesting, and we are reassured by the fact that the findings make biological sense, this study does have limitations—in particular, a lack of test data and the difference of direction of association for PCaa 36:6 in ADNI. Without a full independent test set it is likely that model statistics will be inflated, and therefore the results should be interpreted cautiously. Our preference would have been to split the data into a training and test set; however, the relatively small sample size made this suggestion infeasible. Instead, we choose to use a five-fold CV approach in this study. It is essential to validate this work in independent cohorts of a larger size, for example, in an asymptomatic cohort with high amyloid levels, to reflect the populations eligible for trials such as the A4 trial.^3^ A further issue caused by small sample size is a lack of statistical power. This could be causing the substantial differences in *R*^2^ seen in the continuous NAB analysis and provides further rationale for this work to be replicated in larger cohorts.

A further limitation of this study is the confounding factor of diagnosis: the majority of subjects with high NAB have AD, whereas the majority of subjects with low NAB are diagnosed with fronto-temporal dementia. It is therefore impossible to tell whether the markers we identify here differentiate between high and low NAB or AD and fronto-temporal dementia. Both applications are important and interesting; however, it is vital that we aim to understand this confounding in future studies perhaps through similar analysis in an AD-only cohort.

In further research, targeted metabolite analysis would be beneficial. With an increased annotation, the biological understanding of any findings would grow, potentially deepening our knowledge of the disease mechanism of AD. Metabolite identification using the current methods is time-consuming, often inconclusive and can only be confirmed when the pure standard compounds are available. Further, the presence of annotated data would enable pathway analysis and more ready replication of findings. The data available in ADNI begin to work toward this.

## Conclusion

This study used metabolomic information to predict NAB in subjects from the UCSF AD Research Centre. Five metabolic features identified subjects with high NAB with 72% accuracy. We were able to identify four metabolic features from this panel (PCaa 36:6, PE 39:7 and Anandamide and an isotope) and improve the model further with the addition of FGG protein measures (accuracy=79%). If replicated in large, independent studies, these metabolic features and proteins could form the basis of a blood test with potential for enrichment of amyloid pathology in anti-amyloid trials.

## Figures and Tables

**Table 1:** Cohort demographics

*UCSF*	*Total (*N=*78)*	*Low NAB (*N=*48)*	*High NAB (*N=*30)*	P-*value*
Median NAB SUVR (IQR)^a^	1.3 (0.9)	1.2 (0.1)	2.3 (0.4)	—
Plasma sample median days in storage	1354.5 (560.8)	1400 (432.8)	1247 (835.3)	0.435
Median number of days' difference between sample collection and scan (IQR)	18.5 (69.8)	18.5 (62.3)	16 (101.5)	0.963
Median age (IQR)	65.5 (10.7)	65.8 (9.1)	63.1 (12.7)	0.472
Median MMSE (IQR)	25.5 (6.0)	27 (4.3)	22.5 (9.8)	<0.001

*Scanner type (%)*				
Biograph	9 (11.5)	6 (12.5)	3 (10.0)	>0.999
Siemens	69 (88.5)	42 (87.5)	27 (90.0)	

*Gender (%)*				
Female	32 (41.0)	18 (37.5)	14 (46.7)	0.482
Male	46 (59.0)	30 (62.5)	16 (53.3)	

*APOE* *ɛ4 status (%)*				
0	57 (73.1)	38 (81.2)	18 (60.0)	0.065
1	21 (26.9)	10 (18.8)	12 (40.0)	

*Diagnosis (%)*				
AD	24 (30.8)	2 (4.2)	22 (73.3)	<0.001
FTD	48 (61.5)	42 (87.5)	6 (20.0)	
HC	4 (5.1)	3 (6.3)	1 (3.3)	
MCI	2 (2.6)	1 (2.1)	1 (3.3)	
				
*ADNI*	N=*531*	N=*265*	N=*266*	
Median age (IQR)	75.1 (8.70)	75.8 (8.60)	74.3 (8.78)	0.497

*Gender (%)*				
Female	213 (40.1)	110 (41.5)	103 (38.7)	0.536
Male	318 (59.9)	155 (58.5)	163 (61.3)	
Median years in education (IQR)	16 (4)	16 (4)	16 (4)	0.505

*APOE* ɛ4 status (%)				
0	279 (52.5)	115 (43.4)	164 (61.7)	<0.001
1	252 (47.5)	150 (56.6)	102 (38.3)	
Median MMSE (IQR)	27 (5)	26 (6.75)	28 (4)	0.001

*Diagnosis (%)*				
Other	88 (16.6)	36 (13.6)	52 (19.5)	<0.001
Dementia	157 (29.5)	101 (38.1)	56 (21.0)	
MCI	172 (32.5)	80 (30.2)	92 (34.6)	
HC	114 (21.5)	48 (18.1)	66 (24.8)	

Abbreviations: AD, Alzheimer's disease; ADNI, Alzheimer's disease neuroimaging initiative; FTD, fronto-temporal dementia; HC, healthy control; IQR, interquartile range; MCI, mild cognitive impairment; MMSE, mini mental state exam; NAB, neocortical amyloid burden; SUVR, standardized uptake value ratio; UCSF, University of California, San Francisco.

a This is based on those subjects with SUVR available (*N*=76; low NAB *N*=48; high NAB *N*=28). Kruskal–Wallis *X*^2^ was used to test between high and low groups for continuous demographic variables. Fisher's exact was used to test between high and low groups for categorical demographic variables.

**Table 2:** Multiple metabolic feature analysis

*Continuous NAB models*	R^*2*^	*RMSE*	
Tolerance set (17 metabolic features)	0.07	0.55	
10 Metabolic features	0.05	0.56	
Age and *APOE* status	0.12	0.55	
Tolerance set (17 metabolic features) with FGG	0.57	0.37	
Tolerance set (17 metabolic features) with PPY	0.57	0.38	
Tolerance set (17 metabolic features) with FGG and PPY	0.57	0.37	
FGG and PPY	0.21	0.49	
FGG with *APOE* status and age	0.09	0.53	
PPY with *APOE* status and age	0.01	0.54	
FGG and PPY with *APOE* status and age	0.08	0.52	

*Dichotomized NAB models*	*Accuracy*	*Sensitivity*	*Specificity*
Five metabolic features	0.72	0.65	0.76
Age and *APOE* status	0.58	0.10	0.88
Tolerance set (five metabolic features) with FGG	0.79	0.71	0.84
Tolerance set (five metabolic features) with PPY	0.75	0.58	0.86
Tolerance set (five metabolic features) with FGG and PPY	0.78	0.65	0.89
FGG and PPY	0.72	0.43	0.93
FGG with *APOE* status and age	0.70	0.43	0.90
PPY with *APOE* status and age	0.58	0.26	0.83
FGG and PPY with *APOE* status and age	0.65	0.39	0.86

Abbreviations: FGG, fibrinogen gamma chain; NAB, neocortical amyloid burden; PPY, pancreatic polypeptide; RMSE, root mean square error.

Table shows cross-validated model statistics for continuous NAB.

**Table 3:** Metabolic features included in the multiple metabolic feature models

*Continuous NAB tolerance set model*			*Continuous NAB 10 predictor model*			*Dichotomized NAB model*		
*Mode*	*Median* m/z	*Median retention time (min)*	*Mode*	*Median* m/z	*Median retention time (min)*	*Mode*	*Median* m/z	*Median retention time (min)*
Positive	184.10	2.85	Positive	184.10	2.85	Positive^a^	647.59	10.69
Positive	370.41	11.51	Positive	370.41	11.51	Positive^a^	648.59	10.69
Positive	565.64	18.24	Positive	565.64	18.24	Negative^a^	775.68	16.38
Positive	700.62	17.09	Positive	700.62	17.09	Positive^a^	778.63	14.94
Positive	718.65	17.20	Positive	718.65	17.20	Negative	829.66	16.52
Negative	726.62	18.54	Negative	774.62	18.38			
Positive	755.64	13.93	Negative^a^	775.68	16.38			
Negative	774.62	18.38	Negative	775.63	18.38			
Negative^a^	775.68	16.38	Positive	776.66	18.53			
Negative	775.63	18.38	Negative	829.66	16.52			
Positive	776.66	18.53						
Positive^a^	778.63	14.94						
Positive	784.68	16.19						
Positive	791.68	16.85						
Negative	829.66	16.52						
Positive	903.81	21.38						
Positive	903.86	29.42						

Abbreviation: NAB, neocortical amyloid burden.

a Identified metabolic feature.
