# Supplementary material for: Blood metabolite markers of neocortical amyloid-β burden: discovery and enrichment using candidate proteins
Source: Transl Psychiatry. 2016 Jan 26;6(1):e719–. doi: 10.1038/tp.2015.205 (PMC5068879; doi:10.1038/tp.2015.205)
Supplement: Supplementary Information [file tp2015205x1.docx]

# Supplementary Information

## Pre-processing of metabolite data

The following pipeline was used to process the metabolic feature data once it had been extracted from netCDF files using the R package ‘XCMS’ [1].

1. Metabolic features that eluted before 1 minute or after 35 minutes were removed.
2. Per sample, per metabolic feature outliers were identified as values outside of 6 standard deviations of the mean metabolic feature value and set to missing.
3. Metabolic features not present in at least 80% of samples were removed.
4. Samples that did not have data from at least 80% of metabolic features were removed.
5. Missingness was investigated per batch. In this study batches were the groups of samples split by QC samples.
6. Each metabolic feature was autoscaled (by subtracting the mean value and dividing by the standard deviation) as suggested by van den Berg et al. [2].
7. In order to remove any negative values created by autoscaling (necessary for the remaining processing steps) each value was increased by 6.
8. The distribution of each metabolic feature was tested for normality using the Shapiro-Wilks test.
9. The data was subject to a log base 2 transformation.
10. Batch effects were removed using ComBat [3].
11. Probabilistic principal components analysis (pPCA) was performed to ensure QC samples clustered and to identify any outlier samples.
12. Missing data was imputed using 10 nearest neighbors.

## References

[1]  Smith C, Want E, O’Maille G, Abagyan R and Siuzdak G. XCMS: Processing Mass Spectrometry Data for Metabolite Profiling Using Nonlinear Peak Alignment, Matching, and Identification. *Analytical Chemistry* 2006; **78**(3): 779–787.

[2]  van den Berg R, Hoefsloot H, Westerhuis J, Smilde A and van der Werf M. Centering, scaling and transformations: improving the biological information content of metabolomics data. *BMC Genomics* 2006; **7**(1): 142-157.

[3] Johnson WE, Li C and Rabinovic A. Adjusting batch effects in microarray expression data using empirical Bayes methods. *Biostatistics* 2007; **8**(1): 118–127.
